# Probing the Nanostructure and Arrangement of Bacterial Magnetosomes by Small-Angle X-Ray Scattering

**DOI:** 10.1128/AEM.01513-19

**Published:** 2019-11-27

**Authors:** Sabine Rosenfeldt, Cornelius N. Riese, Frank Mickoleit, Dirk Schüler, Anna S. Schenk

**Affiliations:** a Bavarian Polymer Institute (BPI), University of Bayreuth, Bayreuth, Germany; b Physical Chemistry 1, University of Bayreuth, Bayreuth, Germany; c Department of Microbiology, University of Bayreuth, Bayreuth, Germany; d Physical Chemistry, Colloidal Systems, University of Bayreuth, Bayreuth, Germany; Kyoto University

**Keywords:** small-angle X-ray scattering, SAXS, *Magnetospirillum gryphiswaldense*, magnetosomes, magnetotactic bacteria, magnetic nanostructure

## Abstract

Magnetosomes are membrane-enveloped single-domain ferromagnetic nanoparticles enabling the navigation of magnetotactic bacteria along magnetic field lines. Strict control over each step of biomineralization generates particles of high crystallinity, strong magnetization, and remarkable uniformity in size and shape, which is particularly interesting for many biomedical and biotechnological applications. However, to understand the physicochemical processes involved in magnetite biomineralization, close and precise monitoring of particle production is required. Commonly used techniques, such as transmission electron microscopy (TEM) or Fe measurements, allow only for semiquantitative assessment of the magnetosome formation without routinely revealing quantitative structural information. In this study, lab-based small-angle X-ray scattering (SAXS) is explored as a means to monitor the different stages of magnetosome biogenesis in the model organism Magnetospirillum gryphiswaldense. SAXS is evaluated as a quantitative stand-alone technique to analyze the size, shape, and arrangement of magnetosomes in cells cultivated under different growth conditions. By applying a simple and robust fitting procedure based on spheres aligned in linear chains, it is demonstrated that the SAXS data sets contain information on both the diameter of the inorganic crystal and the protein-rich magnetosome membrane. The analyses corroborate a narrow particle size distribution with an overall magnetosome radius of 19 nm in Magnetospirillum gryphiswaldense. Furthermore, the averaged distance between individual magnetosomes is determined, revealing a chain-like particle arrangement with a center-to-center distance of 53 nm. Overall, these data demonstrate that SAXS can be used as a novel stand-alone technique allowing for the at-line monitoring of magnetosome biosynthesis, thereby providing accurate information on the particle nanostructure.

**IMPORTANCE** This study explores lab-based small-angle X-ray scattering (SAXS) as a novel quantitative stand-alone technique to monitor the size, shape, and arrangement of magnetosomes during different stages of particle biogenesis in the model organism Magnetospirillum gryphiswaldense. The SAXS data sets contain volume-averaged, statistically accurate information on both the diameter of the inorganic nanocrystal and the enveloping protein-rich magnetosome membrane. As a robust and nondestructive *in situ* technique, SAXS can provide new insights into the physicochemical steps involved in the biosynthesis of magnetosome nanoparticles as well as their assembly into well-ordered chains. The proposed fit model can easily be adapted to account for different particle shapes and arrangements produced by other strains of magnetotactic bacteria, thus rendering SAXS a highly versatile method.

## Abstract (precis)

This study explores lab-based small-angle X-ray scattering (SAXS) as a novel quantitative stand-alone technique to monitor the size, shape, and arrangement of magnetosomes during different stages of particle biogenesis in the model organism Magnetospirillum gryphiswaldense. The SAXS data sets contain volume-averaged, statistically accurate information on both the diameter of the inorganic nanocrystal and the enveloping protein-rich magnetosome membrane. As a robust and nondestructive *in situ* technique, SAXS can provide new insights into the physicochemical steps involved in the biosynthesis of magnetosome nanoparticles as well as their assembly into well-ordered chains. The proposed fit model can easily be adapted to account for different particle shapes and arrangements produced by other strains of magnetotactic bacteria, thus rendering SAXS a highly versatile method.

## INTRODUCTION

**FIG 1:**
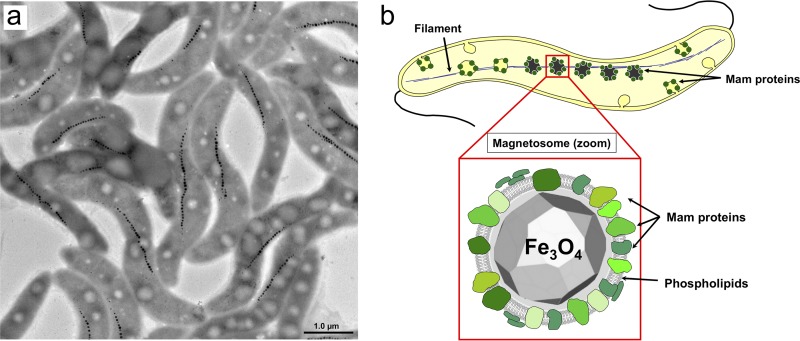
Magnetosome arrangement in *M. gryphiswaldense*. Transmission electron micrograph of wild-type cells illustrating the chain-like particle arrangement (a), as well as a sketch of a single cell with a schematic of a magnetosome consisting of one magnetite core and its surrounding magnetosome membrane (b). The latter contains phospholipids and about 30 different Mam protein species with various functions in the control of magnetosome biomineralization (4).

In order to facilitate their orientation and migration along the Earth’s magnetic field, magnetotactic bacteria (MTB) biomineralize magnetosomes, enabling them to navigate toward favorable habitats in the oxic-anoxic transition zone. In the alphaproteobacterium Magnetospirillum gryphiswaldense (Fig. 1a), these magnetic nanoparticles consist of a cuboctahedral, monocrystalline core of chemically pure magnetite (Fe_3_O_4_), enveloped by a protein-rich biological membrane (magnetosome vesicle) aligned in a linear chain of about 40 particles (Fig. 1b) (1–4). Due to strict control of each step of magnetosome biogenesis, particles with unprecedented characteristics (with respect to crystallinity, particle size, shape, and magnetization) (5, 6) are synthesized. However, despite considerable efforts, the physicochemical processes and steps involved in the biomineralization of well-ordered chains of magnetite nanocrystals are still not understood. This is partially due to a lack of practical lab-scale methods to assess bulk magnetosome formation in growing cells. In addition, magnetosomes are of increasing interest as biogenic magnetic nanoparticles, as they have already been successfully explored in a wide range of potential biotechnical and biomedical applications, including in magnetic hyperthermia (7, 8), as contrast agents (9, 10), or in particle-based immunoassays (11–13). Yet, bioproduction of magnetosome particles depends on highly controlled growth conditions (14, 15) and requires precise and permanent monitoring of bulk magnetosome biomineralization during bacterial cultivation.

Commonly used proxies to estimate magnetosome formation, such as the magnetic orientation of cells (cmag) (16, 17) and their iron content, lack sufficient specificity and do not provide information about the size, structure, and arrangement of particles. In contrast, transmission electron microscopy (TEM) allows the precise determination of particle numbers, sizes, and shapes as well as their chain assemblies. Nonetheless, gathering statistically significant data sets from time-resolved experiments on cultures is very laborious and time-consuming. Furthermore, the analysis is often hindered by preparation or drying artifacts. X-ray radiation-based techniques, such as extended X-ray absorption fine structure (EXAFS) and X-ray absorption near-edge structure (XANES), have already been applied for characterization of magnetite biomineralization; however, quantitative TEM measurements are still necessary to address magnetosome size distribution (18, 19).

As an alternative, small-angle X-ray scattering (SAXS) has been used to assess the structural properties of bacterial magnetosomes (20–22). This technique represents a volume-averaging method to study nanoscale interfaces based on electron density heterogeneities within a sample. SAXS experiments performed on a laboratory-based setup provide access to size, shape, and orientation of nanoscale objects spanning a size range of 1 to 300 nm and are thus ideally suited to study structural parameters and aggregation behavior of intermediates and final products formed during magnetosome biomineralization, as the mature nanoparticles exhibit an average size of about 40 nm (4, 12) and a substantial electron density contrast. During magnetosome biogenesis, the forming particles characteristically undergo a morphological transition leading to changes in the electron density distribution at different growth stages. Such variations in contrast, e.g., evoked by vesicles successively filling up with iron-rich magnetite mineral, are accessible by SAXS and contribute to the scattering pattern in a characteristic way.

Krueger et al. (20) first combined small-angle neutron scattering (SANS) and SAXS to investigate structural parameters of magnetite nanocrystals in magnetotactic bacteria. Due to the restricted data quality of the individual scattering patterns, the results were analyzed by comparing the scattering profiles of samples with low and high iron loadings in the presence and absence of external magnetic fields. The diameters of the magnetosomes biosynthesized during cultivation were estimated to be 40 nm (20). Hoell et al. (21) later investigated M. gryphiswaldense by specialized techniques based on both SANS and SAXS to access the size of the magnetosome membrane and the magnetite crystals as well as their magnetization. More recently, Molcan and coworkers (23) performed a comprehensive structural characterization of magnetosome suspensions from Magnetospirillum magnetotacticum for potential hyperthermia applications by combining a wide range of contrasting analytical techniques, including SAXS. The scattering data in the regime of low values of the scattering vector *q* were fitted by a *q^−x^* power law, where the increase in the exponent *x* was interpreted as a progressing fractal-like aggregation of magnetosomes. Orue et al. (22) focused on the magnetic nanoarchitecture of magnetosomes from *M. gryphiswaldense* but also performed SAXS measurements on highly concentrated bacterial colloids. The authors analyzed the data sets by an indirect Fourier transformation method, which usually relies on additional information regarding the geometry and size of possible aggregate structures. Applying this strategy, Orue and coworkers (22) correlated the first maximum of the pair distance distribution function to the size of one magnetosome and the subsequent maxima to the interparticle distances of adjacent particles. Although comparison with the results from direct visualization by cryo-electron tomography revealed a good agreement of the estimated magnetosome sizes, a 15%-lower value was derived for the correlation distance. However, in all SAXS studies on bacterial magnetosomes, the method was used only on selected specimens as a complementary tool to validate the results obtained from other techniques, such as TEM, electron tomography, or SANS, rather than as a stand-alone analytical method.

In this study, SAXS is explored as a quantitative stand-alone bulk measurement technique allowing time-efficient particle size analysis (in contrast to well-established, but laborious, methods, such as TEM). Despite the detailed structural resolution of magnetosomes, which can be achieved by highly brilliant X-ray beams produced at synchrotron sources (21), limited access as well as usually long distances between production and measurement sites render synchrotron SAXS analysis impractical for fast at-line magnetosome characterization. Accordingly, the present study investigated concentrated cell suspensions of *M. gryphiswaldense* at different growth stages using a lab-scale SAXS system with a rotating copper anode as the X-ray source. This in-house setup offers the advantage of easier access (than with synchrotron measurements) and close proximity to the fermentation facility, thus enabling sampling and process control during magnetosome biosynthesis.

## RESULTS AND DISCUSSION

### Simplified model to describe the magnetosome chain.

As SAXS represents an indirect technique acquiring information in reciprocal space, appropriate models have to be established to derive structural parameters of the sample in real space. Therefore, at first, a model description of the magnetosomes based on both the architecture of individual particles and their arrangement within the cell had to be developed in order to allow for a detailed evaluation of the X-ray scattering data.

**FIG 2:**
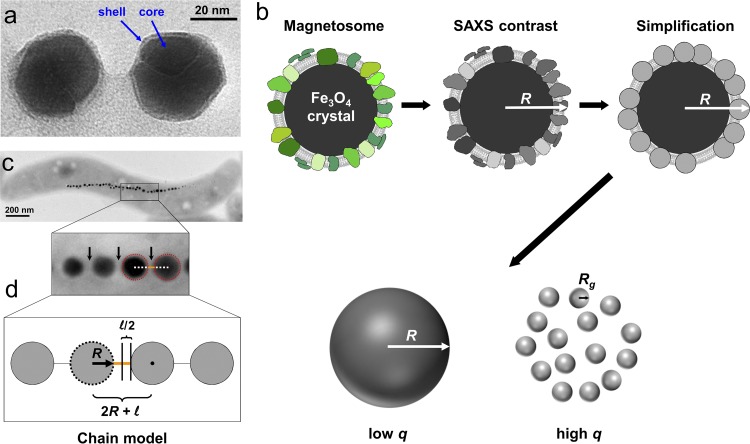
Form factor model of magnetosomes. (a) Transmission electron micrograph of isolated magnetosomes from *M. gryphiswaldense* (photo by F. Mickoleit), exhibiting the characteristic core-shell structure. (b) Sketch of a single magnetosome (as shown in Fig. 1) and the corresponding X-ray contrast model. SAXS is sensitive to the different electron density distributions of the core and the surrounding shell, which consist mainly of magnetite and proteins, respectively. Both contributions are modeled as spheres of different sizes. The resulting contrasts contribute in a characteristic way to specific regions in the scattering data, which consequently contain information on both the overall particle radius, *R*, and the radius of gyration, *R_g_*, of the individual proteins. (c) Chain-like arrangement of magnetosomes within one cell as seen by TEM. Separation distances between single particles are indicated by arrows, while the membranes surrounding the magnetite cores are highlighted by dashed lines. (d) Model of the particle chain shown in panel c. SAXS measurements detect the center-to-center distances of two magnetosomes given by the equation 2*R* + l.

One magnetosome consists of a magnetite crystal, surrounded by a proteinaceous membrane (Fig. 2a), which exerts strict control over the growth of the crystals and their arrangement into well-ordered chains (4, 12). Based on previous TEM imaging of magnetosome chains within *M. gryphiswaldense* (24, 25), magnetosome biogenesis is here considered as the formation of a single linear chain composed of individual core shell particles, which exhibit sharp phase boundaries and are closely packed (Fig. 2c). In the case of a strictly monodisperse system, such an initial intuitive approach would result in an X-ray scattering profile [intensity *I*(*q*) versus *q*] exhibiting pronounced extrema and a characteristic decay in scattering intensity in the regime of low *q* values, but this fails to explain the additional scattering contribution seen in the high *q* range.

Therefore, we propose an alternative model that contains all structural parameters of interest to the here-presented study: the magnetosome particle radius (*R*), the degree of polydispersity, and the distance between neighboring particles (l). For simplification, no indirect Fourier transformation was included in the evaluation. Based on the model presented in Fig. 2b, it can be assumed that the X-ray contrast of a magnetosome is determined mainly by the electron densities of magnetite (∼1,500 electrons/nm^3^) and the attached proteins, for which an electron density in the order of 650 electrons/nm^3^ is estimated by gray-scale analysis of TEM micrographs.

In this model, the magnetite crystal was regarded as a sphere (rather than a facetted object) decorated densely, but randomly, with smaller spheres, simulating protein-crowded particle coverage (Fig. 2b). Furthermore, in order to represent the biological heterogeneity more accurately, a certain polydispersity with respect to the magnetosome radius had to be incorporated into the theoretical description. Under these restrictions, in a multiparticle model, the sharp phase boundary smears out and the magnetosome effectively appears as a simple homogeneous sphere with radius *R*. It has to be considered, though, that this simplified approach does not account for contributions originating from empty vesicles, invaginations, and the cellular matrix, as these do not feature a significant X-ray contrast at the interior. A linear chain of such spheres can then be assumed to describe the geometric arrangement of magnetosomes within the cell (Fig. 2c and d). However, since in a helix-type folding pattern the helical pitch size, i.e., the length of one single turn, most probably exceeds a dimension of 200 nm (24, 25), distinguishing between a helical and a linear chain-like arrangement is beyond the resolution limit of most conventional SAXS setups.

Summarizing the here-presented model, it is suggested that a magnetosome can be simplified as a homogeneous large sphere with a typical size range of 10 to 70 nm surrounded by a 3- to 6-nm-thick proteinaceous phospholipid bilayer that is invaginated from several nonspecific cytoplasmic membrane locations (4, 26). Analysis of the magnetosome subproteome (i.e., the complement of proteins specifically associated with the magnetosome membrane) suggested an unusually crowded protein composition on the membrane and a tight packing with transmembrane domains of integral proteins (27). Depending on their size and folding (the numbers of transmembrane helices and extramagnetosomal domains), the magnetosome proteins can be modeled as small objects (spheres) typically in the size range of 4 to 10 nm (28). For simplicity, a coiled (spherical) supramolecular arrangement/folding pattern is assumed.

In the regime of small *q* (corresponding to large dimensions in real space), the X-ray scattering pattern is insensitive to the fine details of the protein shell. Hence, while the proteins significantly increase the contrast of the shell, they are not distinguishable as separate small spherical objects. Consequently, the SAXS profile is dominated by the overall size of the particles, and data analysis in the lower *q* range yields the magnetosome radius, *R*, i.e., the outer radius determined by the thickness of the magnetosome membrane (shell). However, in the regime of high *q* (corresponding to smaller dimensions in real space), X-ray scattering detects fluctuations in the electron density distribution on a smaller length scale and therefore is sensitive to finer structural motives, such as the proteins themselves.

### Calculation of the scattering function.

The intensity [*I*(*q*)] is determined by the square of the amplitude of the scattered wave, which, in turn, is given by equation 1 in the case of a particle with volume *V_p_* and an excess electron density distribution, Δ*ρ*(*r*).

(1) $$A\left(q\right)\;=\;4\pi \underset{{}_{V_{p}}}{\int }\Delta \rho \left(r\right)\frac{\sin\;\left(\mathrm{qr}\right)}{\mathrm{qr}}r^{2}dr$$

where *A*(*q*) is amplitude. For a system of monodisperse spheres with radius *R*, equation 1 can be expressed as a function of the particle radius according to equation 2.

(2) $$A\left(q\right)\;=\;\frac{4\pi R^{3}}{3}\frac{3\left[\sin\;\left(\mathrm{qR}\right)\;-\;\mathrm{qR}\;\cos\;\left(\mathrm{qR}\right)\right]}{{\left(\mathrm{qR}\right)}^{3}}$$

In order to calculate the intensity for polydisperse systems composed of different particle species with size *i*, the squared amplitudes, $A_{i}^{2}\left(q\right)$, have to be added up and weighted by their respective number density, *N/V_p_*.

### X-ray scattering analysis of magnetosome particles.

The scattering intensity, *I*(*q*), originating from noninteracting small and large spheres is given by the corresponding amplitudes, according to equation 3 (29):

(3) $$I\left(q\right)\;=\;A_{\text{big}}^{2}\left(q\right)\;+\;\underset{i}{\sum }A_{\text{small}}^{2}\;+\;\underset{i}{\sum }A_{\text{big}}\;(q)\;A_{\text{small}}\;\left(q\right)\frac{\sin\;\left(\mathrm{qd}\right)}{\mathrm{qd}}\;+\;2\underset{i\neq j}{\sum }A_{\text{small},i}\;(q)\;A_{\text{small},j}\;\left(q\right)\frac{\sin\;\left(qd_{\mathrm{ij}}\right)}{qd_{\mathrm{ij}}}$$

where *d* denotes the distance between the centers of gravity of the large and the small spheres and *d_ij_* represents the mutual distances between small spheres. The first two terms are equal to the intensities of noninteracting spheres of the corresponding size (equation 2). In the case of large spheres, the first term decays rapidly with *I*(*q*) being ∼*q*^−4^ and therefore does not contribute significantly to the scattering intensity at high values of *q* (see Fig. S1 in the supplemental material), whereas the second term associated with the small spheres (big amount) plays the dominant role. Due to polydispersity in real-world systems, the factor [sin(*qd*)/(*qd*)] cancels out the third term at high *q*. Concomitantly, the average over the fourth term vanishes as distance, *d_ij_*, varies randomly. As discussed above, SAXS represents a low-resolution method at low scattering angles. As a consequence, term 2, i.e., the sum of the contribution of the small spheres, can alternatively be described as an additional contribution to the electron density of the entire large sphere. The protein decoration may then be regarded as independent small-density fluctuations; thus, according to scattering theory, the intensities of these fluctuations will add up to describe the scattering of a much larger object with high contrast (here corresponding to the magnetosome).

Therefore, particularly in view of potential applications in on-line analysis of magnetosome biosynthesis, the SAXS data analysis of these biogenic particles can be simplified by assuming that the total intensity is modeled by a term corresponding to the large spheres and an additional contribution originating from the small objects, e.g.,

(4) $$I\left(q\right)=I_{\text{sphere}}\left(q\right)+I_{\text{small}}\left(q\right)$$

**FIG 3:**
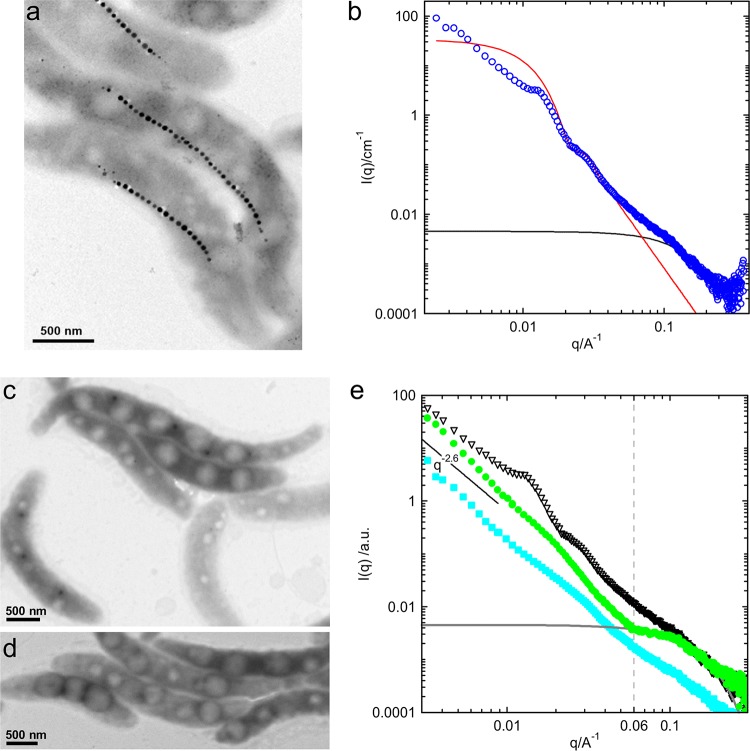
Comparison of magnetosome-free and -producing cells of *M. gryphiswaldense*. (a) Transmission electron micrograph of representative magnetosome-producing cells grown under anaerobic conditions. (b) Scattering patterns obtained from cell suspensions (blue circles) shown in panel a. Additionally, the calculated scattering patterns of small spherical objects according to equation 5 (solid black line) and polydisperse spheres according to equation 2 (red line) are shown. The spheres exhibit a mean radius of 19 nm with a standard deviation of 17% (Gaussian distribution). (c and d) Transmission electron micrographs of representative, magnetite-free cells of *M. gryphiswaldense*. (e) Scattering patterns of the corresponding *M. gryphiswaldense* cell suspensions. The curves are presented with a vertical shift for better visibility. Open symbols, magnetite-producing *M. gryphiswaldense* cells as shown in panel a; filled symbols, magnetite-free cells (green or blue symbols correspond to cells shown in panel c or d, respectively). Additionally, the calculated scattering profile of the small particles is provided for comparison (gray line).

Irrespective of the particle morphology, the scattering intensity *I*(*q*) for a dilute solution of small objects is given by Guinier’s law

(5) $$I_{\text{small}}\left(q\right)\;\approx \;\Delta \rho^{2}cM_{w}\;\exp\;\left(-\frac{R_{g}^{2}}{3}q^{2}\right)$$

where Δ*ρ* denotes the electron density contrast of the small spheres, *c* the weight concentration, *M*_w_ the molecular weight, and *R_g_* the radius of gyration. For the subsequent analysis, it is important to note that *I*_small_(*q*) contributes mainly to the scattering intensity for a *q *of >0.05 Å^−1^ (Fig. 3) and can be approximated by Guinier’s law, which allows us to perform profile fitting based on a minimum of adjustable parameters.

With the terms for the evaluation of magnetosome and protein radius at hand, anaerobically grown, magnetosome-producing cells were analyzed by TEM and SAXS (Fig. 3). In this experiment, the radius of the magnetosome is derived from the position of the first minimum (*q *≈ 0.02 Å^−1^) as *R *equaling 19 nm ± 17% (Gaussian size distribution). The radius of gyration of the small objects (proteins) is determined as 1.7 nm and correlates with radius *R* of a solid sphere via equation 6,

(6) $$R_{g}\;=\;\sqrt{\frac{3}{5}}R$$

yielding an *R *equal to 2.2 nm. This value is comparable to the general dimensions expected for the coiled proteins based on TEM imaging (28, 30).

Hence, it can be concluded that within the framework of the aforementioned assumptions, the scattering pattern is well described by the proposed model for *q* > 0.015 Å^−1^.

To further test this concept, magnetosome-free cells of *M. gryphiswaldense* (aerobically grown), which are characterized by the lack of magnetite crystals but still contain in some cases (empty) membrane vesicles (26), were analyzed. For this purpose, SAXS data obtained from magnetite-free and magnetite-producing cells (Fig. 3c to e) were compared. Intriguingly, both magnetite-free systems (compare the blue and green curves in Fig. 3e) do not exhibit the characteristic oscillations observed for producing cells in the mid *q* range (*q* ∈ [0.08 Å^−1^, 0.03 Å^−1^]) but instead show a similar linear decay at low *q* (*q *< 0.01 Å^−1^). In contrast, at high values of *q*, where small nanostructural features dominate the curve, the scattering profiles of magnetosome-deficient cells and magnetosome-producing bacteria (i.e., cells that synthesize magnetite cores enveloped by the magnetosome membrane) are almost identical. In some cases, here exemplified by the green curve, even a clear kink is observed (*q *≈ 0.06 Å^−1^), indicating the presence of a huge amount of very well defined small objects, such as proteins, which are highly abundant in the cells.

These observations strongly support the assumption that the total scattering intensity of one magnetosome (form factor scattering) can be deconvoluted into partial intensities assigned to large and small spherical objects, *I*_sphere_(*q*) and *I*_small_(*q*). Based on these results, one can in principle even consider the magnetosome-deficient phenotype as background when investigating the scattering observed for anaerobically grown *M. gryphiswaldense* cells, which biosynthesize magnetosomes. However, while there were no phenotypical differences between the two specimens of magnetite-free bacteria identified by TEM (Fig. 3c and d), their corresponding SAXS profiles clearly showed a deviation, particularly in the regime of high *q* (Fig. 3e, blue and green curves), which indicates a substantial biological variability in these systems and thus complicates the identification of a suitable background curve.

The assembly of the magnetosomes into ordered chains is the major reason for the deviation between the experimentally observed scattering intensity at low *q* (Fig. 3e, black curve) and the here-applied fit model. Due to the high degree of order (aligned magnetosomes), Bragg’s law is suited to determine interparticle separation distances, such that a correlation length of approximately 47 nm can be determined based on the position of the first maximum (*q *≈ 0.0134 Å^−1^).

### Determination of structural magnetosome assembly into chains.

Chain assembly results from the coordinated interplay of collaborative magnetic interactions, which occur naturally between magnetite nanocrystals, in combination with active protein-mediated bio-assembly mechanisms (31, 32). Cellular structures stabilize the string of magnetic dipoles, thereby avoiding a magnetic-moment-induced collapse into rings or irregular clusters (33). In this geometric arrangement, the actin-like MamK filament anchors the magnetosome chain and generates an intracellular compass needle to maximize the cell response to even low magnetic fields experienced under environmental conditions. The pole-to-midcell treadmilling growth of MamK filaments provides the key driving force for magnetosome movement (34).

Typically, wild-type cells of *M. gryphiswaldense* synthesize 1 to 2 magnetosome chains that extend across approximately two-thirds of the length of the cell and are located at midcell. An average chain is composed of ≈20 to 40 particles with a mean center-to-center distance of about 50 to 60 nm (22, 24, 25).

According to the decoupling approximation, the total scattering intensity is proportional to the product of the scattering originating from one particle (form factor) and the scattering due to interparticle interactions (structure factor). The structure factor, *S*(*q*), is related to the probability distribution function of interparticle distances, *g*(*r*), and can be expressed according to equation 7 for isotropic systems.

(7) $$S\left(q\right)\;=\;1+\int_{0}^{\infty }4\pi r^{2}\left[g\left(r\right)-1\right]\frac{\sin\;\left(\mathrm{qr}\right)}{\mathrm{qr}}dr$$

In an ideal solution featuring no position or orientation correlations, *S*(*q*) can be assumed to equal 1, whereas ordered crystal-like particle arrangements exhibit an *S*(*q*) structure factor of >2.85 according to the Hansen-Verlet criterion (35). The here-presented approach to consider the data set obtained from the magnetite-deficient bacteria as the background signal for the scattering of magnetosome-containing cells affords the possibility of experimentally estimating the particle-particle interaction via the experimental structure factor *S*(*q*), which is obtained by dividing the scattering intensity of the magnetite-containing sample by the intensity of the respective magnetite-deficient sample.

**FIG 4:**
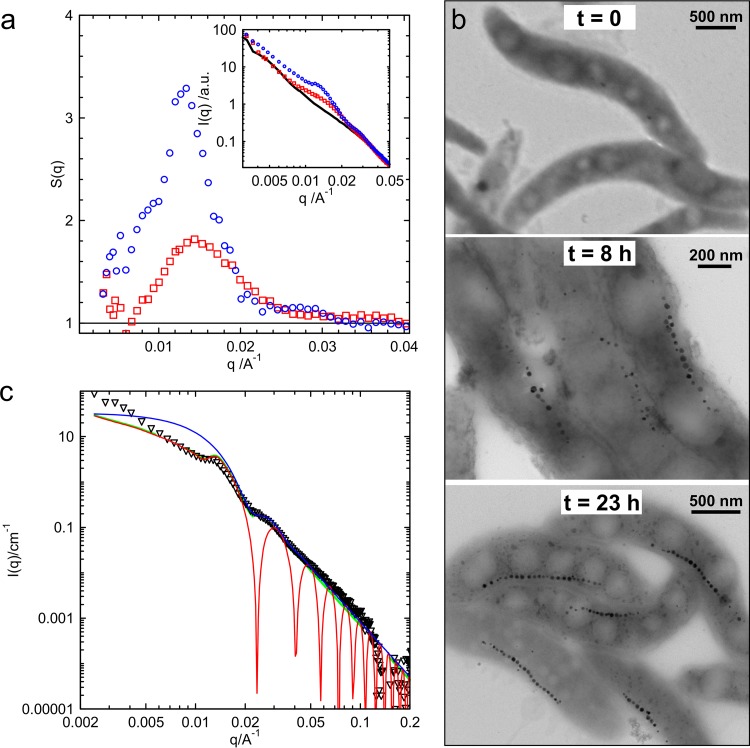
Scattering patterns and transmission electron micrographs of *M. gryphiswaldense* at different sampling times during cultivation. (a) Patterns at the start of production (0 h, black line, no magnetite due to growth conditions), after 8 h (red squares), and after 23 h (blue circles). (b) Corresponding transmission electron micrographs. (c) The scattering of fully matured cells [open triangles, after subtraction of the scattering contribution, *I*_small_(*q*)] is compared to the form factor scattering of polydisperse, noninteracting spheres (thin blue line, *R *= 19 ± 3 nm, Gaussian distribution) and the scattering of 5 monodisperse spheres arranged in a linear chain (thin red line, *R *= 19 nm, *l *= 12 nm). A model allowing for polydispersity in the system is represented by the green line. a.u., arbitrary units; t, time.

Next, magnetosome biomineralization and chain formation during cell growth were investigated. Therefore, samples were drawn at different production time points and subsequently analyzed by TEM and SAXS (Fig. 4). Using the scattering data obtained at time zero as background (Fig. 4a, black line), the experimental structure factors were determined as follows: *S*(*q*) was equal to 1 at the start, *S*(*q*) was approximately equal to 1.8 after 8 h, and *S*(*q*) was approximately equal to 3.2 after 23 h of biomineralization; this corresponds to the degree of particle interaction and is accompanied by the evolution from a nonordered to a highly ordered spatial correlation of the magnetite cores into a single chain. In support of the here-proposed theoretical model, the extracted structural parameters are in very good agreement with TEM results (Fig. 4b), which clearly demonstrate the one-dimensional crystalline order of magnetosomes after 23 h.

Such an ordered nanoparticle array resembles a chain as described by a linear pearl model (36), which is based on the form factor for *N_S_* spheres with radius *R* linearly joined by short strings of length l (Fig. 2) (the thickness of the strings is not taken into account). The scattering intensity, *I*(*q*), for such an assembly is given by equation 8.

(8) $$I\left(q\right)\;=\;\frac{N}{V}\left\{{\left(\Delta \rho \;V_{N_{S}\text{pearls}}\right)}^{2}\left[N_{S}+2\overset{N_{S}}{\underset{n=1}{\sum }}\left(N_{S}-n\right)\frac{\sin\;\left(\mathrm{nq}l\right)}{\mathrm{nq}l}\right]{\left[3\frac{\sin\;\left(\mathrm{qR}\right)-\mathrm{qR}\;\cos\;\left(\mathrm{qR}\right)}{{\left(\mathrm{qR}\right)}^{3}}\right]}^{2}\right\}$$

This consideration accounts for an increase in the scattering intensity on length scales of the order of *q*(2*R +* l) being approximately equal to 2π*k*, where *k* is an integer.

The scattering data in Fig. 4c were obtained from fully mature *M. gryphiswaldense* cells after subtraction of the scattering contribution, *I*_small_(*q*). For comparison, the theoretical scattering curve of spheres with a radius of 19 nm (±17%, Gaussian size distribution) and the intensity profile of 5 monodisperse spheres arranged in a line with a center-to-center distance of *2R* + l equal to 50 nm are depicted. The modeled interaction distance of 50 nm is in very good agreement with the oscillation pattern in the scattering data arising from interparticle interactions (first maximum observed at a *q *approximately equal to 0.013 Å^−1^) and most importantly is confirmed by TEM analysis, which reveals a separation distance of *2R* + l equaling 53 ± 6.0 nm.

### Conclusions.

Routinely used methods to monitor magnetosome biosynthesis, such as cmag (16, 17), iron content measurement, and EXAFS (18), as well as TEM, can often only provide a semiquantitative assessment of magnetosome structure and content within the investigated cells, thus rendering them comparably impractical for on-line process control and consequently generating a strong demand for efficient quantitative bulk methods. Therefore, the present study introduces SAXS as a nondestructive (i.e., leaving membranes and proteins intact) stand-alone tool for process monitoring during magnetosome production.

In order to model the here-obtained scattering profiles, magnetosomes were considered as polydisperse spheres arranged into a single linear chain, which is reflected in the scattering signal at low *q*, whereas the cell’s densely packed protein background dominates the signal at high *q*. Based on these simplifications, a linear pearl model without indirect Fourier transformation was applied to describe concentrated cell suspensions grown under anaerobic (magnetosome-producing) and aerobic (magnetosome-free) conditions. The linear pearl model based on five polydisperse spheres turned out to be best suited for describing the experimental data, yielding magnetosome sizes that could essentially be confirmed by TEM analysis. In conclusion, this study demonstrates that SAXS measurements performed on a laboratory instrument can be used as a convenient, readily accessible, and potentially even automated method to monitor bulk magnetosome biosynthesis in growing cells and cultures. Most importantly, SAXS is a volume-averaging, quantitative technique providing statistically accurate information on size, shape, and arrangement of nanostructural motives in a sample. Additionally, the lab-scale in-house equipment employed in this study enables at-line analysis of magnetosome biomineralization and prospectively even on-line process control during fermentation. Therefore, SAXS has the potential to complement and partially replace laborious and time-consuming quantitative TEM analyses, thereby allowing for routine magnetosome characterization. Applying the here-proposed model, structural parameters, such as the dimensions of the inorganic crystal core and the protein-rich membrane, as well as the arrangement and assembly of the magnetosomes, can be extracted with high statistical accuracy. In view of a more general applicability to different species and their magnetosome mutants grown under different conditions, the model can easily be adapted and modified to account for different particle shapes and arrangements within the cell, e.g., by implementing form factors for nonspherical scattering objects. The general contrast situation with respect to the electron density heterogeneities within the sample (i.e., Fe-containing mineral versus organics) should not be affected by the specific strain of bacteria used, thus rendering the proposed method a highly versatile tool for elucidating further details in magnetosome formation.

## MATERIALS AND METHODS

### Cultivation of *M. gryphiswaldense*.

The *M. gryphiswaldense* wild type was grown either aerobically or anaerobically at 28°C in a BioFlo 320 Eppendorf Bioprocess fermentor system (Jülich, Germany) using a modified large-scale medium (14). Cells were harvested by low-spin centrifugation (7,000 rpm, 10 min, 4°C, Avanti J-26SXP; Beckman Coulter, USA). Cell pellets were washed and resuspended in 10 mM 2-[4-(2-hydroxyethyl)piperazin-1-yl]ethanesulfonic acid (HEPES), pH 7.0, before transferring the suspension into glass capillaries (∅ = 1 mm; Hilgenberg, Germany) for further analysis.

### Structural characterization of magnetosome nanoparticles. (i) TEM.

For transmission electron microscopy (TEM) analysis of whole cells, specimens were directly deposited onto carbon-coated copper grids. TEM imaging was performed on a JEOL JEM-1400+ transmission electron micrograph (Freising, Germany) operated at an acceleration voltage of 80 kV. Images were recorded with a Gatan Erlangshen ES500W charge-coupled device (CCD) camera. Average particle sizes were measured by data processing using the ImageJ v1.52i software package.

### (ii) SAXS.

Nanostructure analysis of bacterial magnetosomes was performed on highly concentrated bacterial suspensions under ambient conditions using a Double Ganesha AIR system (SAXSLAB, Denmark). Monochromatic radiation with a wavelength (λ) of 1.54 Å was generated by a rotating anode source (Cu; MicroMax 007HF; Rigaku Corporation, Japan). All data sets were recorded with a position-sensitive detector (Pilatus 300K; Dectris) placed at different distances from the sample to cover a wide range of scattering vectors, *q*, with 0.002 Å^−1^ < *q *< 0.5 Å^−1^, where *q* is given as

(9) $$q\;=\;\left|\overset{\to }{q}\right|\;=\;\frac{4\pi }{\lambda }\sin\;\left(\frac{\theta }{2}\right)$$

with λ representing the wavelength of the incident beam and θ the scattering angle.

The two-dimensional scattering patterns were converted into one-dimensional intensity profiles of *I*(*q*) versus *q* by radial averaging and subsequently normalized to the intensity of the incident beam, the sample thickness, and the accumulation time. Background correction was performed by subtracting the signal of a glass capillary (∅ = 1 mm; Hilgenberg, Germany) filled with HEPES buffer. The software SasView 4.2 was used for data analysis.

## Supplementary Material

Supplemental file 1
