# Supplementary material for: Probing the Nanostructure and Arrangement of Bacterial Magnetosomes by Small-Angle X-Ray Scattering
Source: Appl Environ Microbiol. 2019 Nov 27;85(24):e01513-19. doi: 10.1128/AEM.01513-19 (PMC6881800; doi:10.1128/AEM.01513-19)
Supplement: Supplemental file 1 [file AEM.01513-19-s0001.pdf]

Supporting Information

Probing the nanostructure and arrangement of bacterial magnetosomes by small-angle x-ray scattering

Sabine Rosenfeldt[# 1,2], Cornelius N. Riese[# 3], Frank Mickoleit[3], Dirk Schüler[3], Anna S. Schenk*[1,4]

[#] *These authors contributed equally to this work*

[1]*Bavarian Polymer Institute (BPI),* [2]*Physical Chemistry 1,* [3]*Dept. Microbiology,* [4]*Physical Chemistry - Colloidal Systems; University of Bayreuth, D-95447 Bayreuth*

*Corresponding author:

Jun.-Prof. Dr. Anna S. Schenk, Physical Chemistry - Colloidal Systems, University of Bayreuth, D-95447 Bayreuth; E-Mail: anna.schenk@uni-bayreuth.de; Tel.: +49-921-55-3915

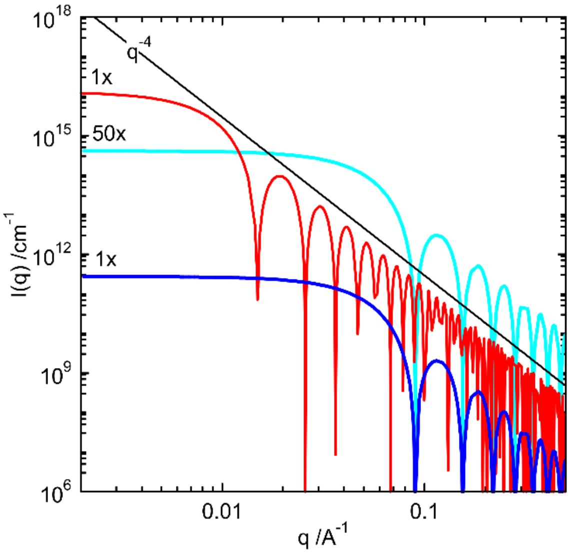

Fig.S1 Theoretical scattering profiles calculated for an individual large sphere (radius R = 30 nm, red curve) as compared to either one single smaller sphere (radius R = 5 nm, blue curve) or an ensemble of 50 smaller spheres (radius R = 5 nm, cyan curve)

The plot illustrates the characteristic theoretical scattering profiles of monodisperse spheres with identical contrast. The intensities exhibit pronounced minima, which determine the respective radii of the spheres. In agreement with the form factor expected for spherical scattering objects, the intensity decays with $I(q) \sim q^{-4}$ at large q (i.e. $qR >> 1$). In a multi-particle system, the scattering contributions of the individual scatterers add up such that scattering intensity of N = 50 spheres with radius R = 5 nm is significantly higher at high q (q > 0.1 Å^{-1}) than the signal originating from one larger sphere with R = 50 nm in this region.
